# Measurement of the levels of leptin, BDNF associated with polymorphisms *LEP* G2548A, *LEPR* Gln223Arg and *BDNF* Val66Met in Thai with metabolic syndrome

**DOI:** 10.1186/1758-5996-6-6

**Published:** 2014-01-21

**Authors:** Kanjana Suriyaprom, Rungsunn Tungtrongchitr, Kittisak Thawnasom

**Affiliations:** 1 Faculty of Medical Technology, Rangsit University, Paholyothin Road, Pathumthani 12000, Thailand; 2 Department of Tropical Nutrition & Food Science, Faculty of Tropical Medicine, Mahidol University, 420/6 Rajvithi Road, Rajthevee, Bangkok 10400, Thailand

**Keywords:** Leptin, BDNF, Gene polymorphisms, Metabolic syndrome

## Abstract

**Background:**

Metabolic syndrome is a cluster of metabolic risk factors including dyslipidemia, impaired glucose tolerance, hypertension and central obesity. BDNF (Brain-derived neurotrophic factor) and leptin have been implied in the energy homeostasis. The purposes of this study were to examine concentrations of leptin, BDNF and biochemical parameters in metabolic-syndrome subjects and healthy controls, and also to search for associations of leptin gene *(LEP)* G2548A, leptin receptor gene *(LEPR)* Gln223Arg, and BDNF gene *(BDNF)* Val66Met polymorphisms with leptin levels, BDNF levels and metabolic syndrome among Thais.

**Methods:**

The case-controlled design was performed using 322 Thai volunteers (160 metabolic-syndrome subjects; 162 controls) during the health screening program. Metabolic syndrome was assessed by using the modified National Cholesterol Education Program, Adult Treatment Panel III criteria. The levels of leptin, BDNF, insulin, glucose and lipids were measured in samples. Genotyping of *LEP* G2548A, *LEPR* Gln223Arg and *BDNF* Val66Met was carried out using polymerase chain reaction-restriction fragment length polymorphism technique.

**Results:**

Serum leptin levels were significantly higher in the metabolic-syndrome group than the control group (p < 0.01), but the BDNF difference between them was not significant. Significant associations of *LEPR* Gln223Arg polymorphism were found with leptin and glucose levels (p < 0.05), after adjusting for potential covariates. This *LEPR* polymorphism in the metabolic-syndrome group was also significantly more frequent than in the control group (p < 0.05). However, other gene polymorphisms, *LEP* G2548A and *BDNF* Val66Met, showed no significant relationship with leptin levels, BDNF levels or metabolic syndrome.

**Conclusion:**

These findings suggest leptin levels are linked with metabolic syndrome. *LEPR* Gln223Arg polymorphism impacted leptin concentrations, and this gene polymorphism may influence susceptibility to metabolic syndrome among Thais.

## Introduction

Metabolic syndrome (MS), which involves abdominal obesity, dyslipidemia, hypertension, and glucose tolerance represents one of the world’s most challenging health problems, and is an important risk factor for type 2 diabetes and cardiovascular diseases [1]. Leptin, an adipocytokine, is a key metabolic regulator and acts to reduce food intake, increase energy expenditure, and modulate immune and inflammatory responses by binding and activating the leptin receptor [2]. Serum leptin is proportional to body adiposity [3]. There are several studies indicating links of leptin gene (*LEP*) and leptin receptor gene (*LEPR*) variations with obesity [4], insulin resistance [5], and type 2 diabetes [6]. The gene polymorphisms of *LEP* G2548A (rs7799039: guanine > adenine) and *LEPR* Gln223Arg (rs1137101: glutamine > arginine) are studied widely because *LEP* G2548A has been associated with leptin production and secretion [7], and *LEPR* Gln223Arg has been associated with impaired signaling capacity of the leptin receptor [8]. However, evidence for an association of *LEP* G2548A and *LEPR* Gln223Arg with leptin concentrations and MS is inconsistent [9-11]. Moreover, the relationship of *LEP* G2548A and *LEPR* Gln223Arg gene polymorphisms with MS among Thais has not been investigated to date.

Brain-derived neurotrophic factor (BDNF) is a member of the neurotrophic factor family and is one of the molecules that mediate the function in the control of energy balance. The associations of BDNF and leptin with functions involved in energy homeostasis have been studied [12]. Previous studies have shown links between BDNF concentrations and obesity and diabetes [13,14]. The most common BDNF gene (*BDNF)* polymorphism, Val66Met (rs6265: valine > methionine), affects intracellular trafficking of pro-BDNF, and also decreases secretion and production of the mature BDNF with the Met allele [15]. However, previous studies have shown inconsistent results in relation to BDNF concentrations in MS subjects [16,17], and information on *BDNF* Val66Met gene polymorphism and MS is scarce. Therefore, the aims of this study were to examine concentrations of leptin, BDNF and biochemical parameters in MS subjects and healthy controls, and to search for links of *LEP* G2548A, *LEPR* Gln223Arg and *BDNF* Val66Met gene polymorphisms with leptin levels, BDNF levels and MS among Thais.

## Methods

Following approval by the Ethics Committee of Rangsit University (RSEC No.016/53), Thailand and in accordance with the Declaration of Helsinki, this case-control study enrolled 322 Thai volunteers aged between 24 to 64 years and living in urban and suburban residential areas of Bangkok, Thailand. Among them, 162 healthy controls (86 male, 76 female) and 160 MS subjects (73 male, 87 female) were chosen during the health screening program among check-up subjects in March 2011- February 2012 and the statistical power in our sample size calculation was 80% at alpha =0.05. A physical examination and medical history check on all subjects was performed. Subjects with a history of liver, kidney and cardiovascular diseases were excluded from the study. Metabolic syndrome was defined using the modified National Cholesterol Education Program/Adult Treatment Panel III (NCEP/ATP III) criteria [18]. The new cut-off for waist circumference in the Asia-Pacific Region, instead of the original cut-off in the ATP III criteria, was used. Adoption of the new cut-off point for fasting plasma glucose criterion has already been reported (> 100 mg/dl). The modified NCEP/ATP III definition required at least three of the following: raised triglyceride (TG) levels: > 150 mg/dl, reduced high-density lipoprotein cholesterol (HDL-C): < 40 mg/dl in men, and < 50 mg/dl in women, raised fasting plasma glucose: > 100 mg/dl, raised waist circumference (WC): > 90 cm in Asian men and > 80 cm in Asian women or raised blood pressure: systolic blood pressure > 130 mmHg or diastolic blood pressure > 85.

### Anthropometric measurements

The body weight of each subject was measured using a carefully calibrated beam balance (Detecto®, Cardinal Detecto Scale Manufacturing, MO). Height was measured using a vertical measuring rod. Body mass index (BMI) was conventionally expressed as weight (kg)/height (m^2^). Waist and hip circumferences were measured. Waist and hip circumferences were calculated for waist and hip circumference ratio. Furthermore, this study had also determined triceps skin-fold thickness (TSF) and arm circumference (AC) because TSF was suggested as the simple predictor of body density and hence percentage total body fat [19] and AC measurement could reflect adult nutritional status as defined by BMI [20]. The upper arm length midpoint mark was used to measure TSF and AC. The TSF was measured by using the Holtain Skinfold Caliper. Blood pressure (BP) was measured by a nurse after 5 to 10 minutes’ rest in the sitting position.

### Laboratory measurements

10 ml of venous blood was taken from subjects in the morning after overnight fast. Glucose, TG, HDL-C levels were measured using enzymatic methods by DADE Dimension AR®. Leptin and insulin levels were measured using a radioimmunoassay kit from Linco Research (St Louis, USA). Serum BDNF levels were determined using an ELISA protocol, according to the manufacturer’s instructions (DBD00; R & D Systems, Europe).

### Polymerase chain reaction-restriction fragment length polymorphism (PCR-RFLP) technique

DNA was extracted from EDTA-treated whole blood by FlexiGene DNA kit (Qiagen, Hilden, Germany). Genotyping of *BDNF* Val66Met, *LEP* G2548A and *LEPR* Gln223Arg was carried out using PCR-RFLP assay. DNA fragments were amplified by PCR (PE Applied Biosystems). For analysis of the *BDNF* Val66Met polymorphism, the following primers were used:

Forward primer – 5′-ATCCGAGGACAAGGTGGC-3′

Reverse primer – 5′-CCTCATGGACATGTTTGCAG-3′

A 50 μl PCR reaction was performed according to the protocol described by Matsushita *et al.*[21]. For analysis of the *LEP* G2548A polymorphism, the following primers were used:

Forward primer – 5′-TTTCTGTAATTTTCCCGTGAG-3′

Reverse primer – 5′-AAAGCAAAGACAGGCATA AAAA-3′

A 50 μl PCR reaction was conducted, according to the protocol described by Boumaiza *et al.*[10]. For analysis of the *LEPR* Gln223Arg polymorphism, the following primers were used:

Forward primer – 5′-ACCCTTTAAGCTGGGTGTCCCAAATAG-3′

Reverse primer – 5′-AGCTAGCAAATATTTTTGTAAGCAATT-3′

**Figure 1:**
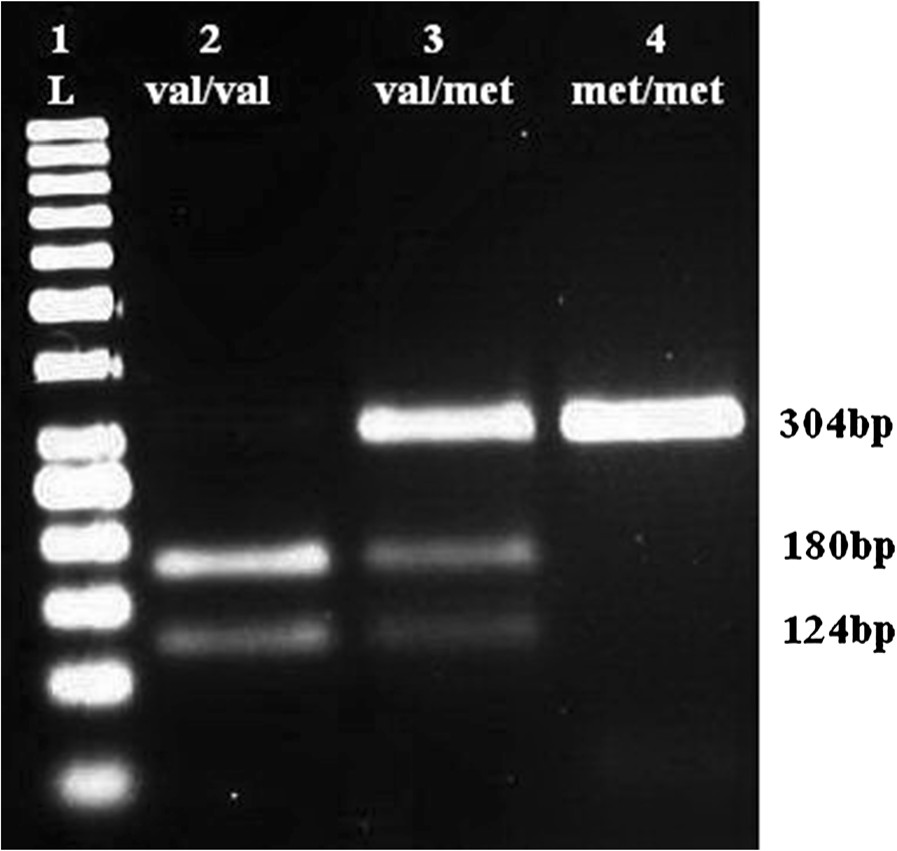
**Restriction fragments length polymorphism (RFLP) distribution pattern of *BDNF *Val66Met polymorphism.** Lane 1 = ladder (50 bp), Lane 2 = Val/Val (124 and 180 bp), Lane 3 = Val/Met (304, 180 and 124 bp), Lane 4 = Met/Met (304 bp).

**Figure 2:**
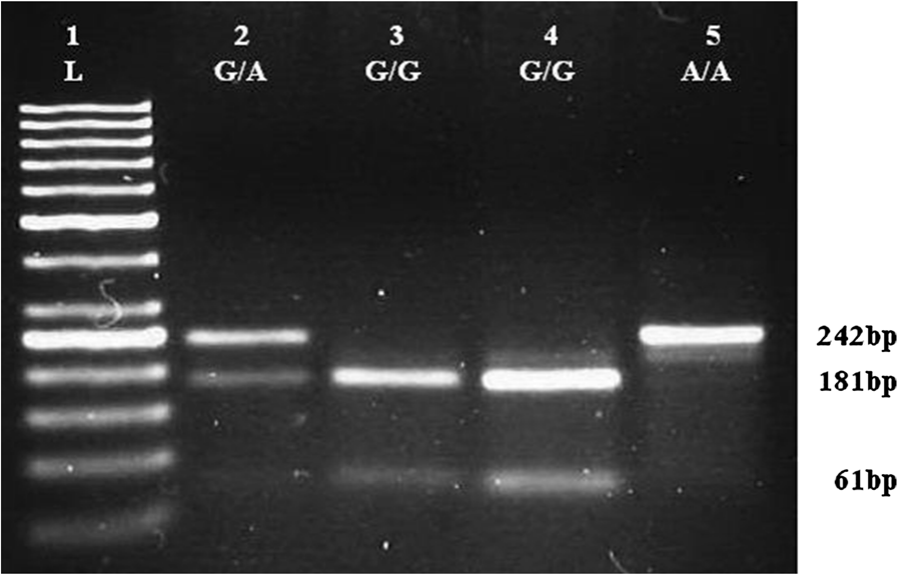
**Restriction fragments length polymorphism (RFLP) distribution pattern of *LEP *G2548A polymorphism.** Lane 1 = ladder (50 bp), Lane 2 = G/A (242,181, and 61 bp), Lane 3-4 = G/G (181 and 61 bp), Lane 5 = A/A (242 bp).

**Figure 3:**
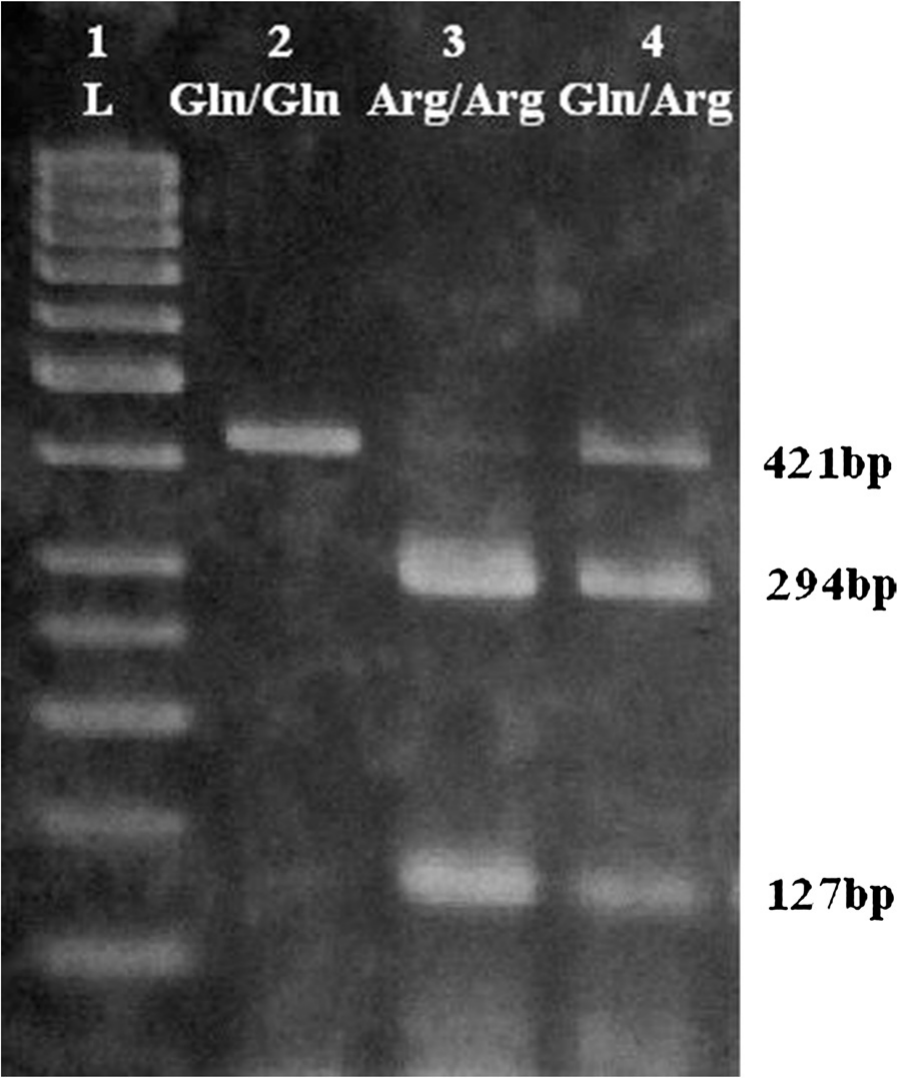
**Restriction fragments length polymorphism (RFLP) distribution pattern of *LEPR *Gln223Arg polymorphism.** Lane 1 = ladder (50 bp), Lane 2 = Gln/Gln (421 bp), Lane 3 = Arg/Arg (294 and 127 bp), Lane 4 = Gln/Arg (421, 294 and 127 bp).

A 50 μl PCR reaction was conducted, according to the protocol described by Duarte *et al.*[4]. PCR products (304 bp for *BDNF*, 242 bp for *LEP* and 421 bp for *LEPR*) were detected on 2% agarose gel containing ethidium bromide. Aliquots of the PCR products were digested with 8 U *PmlI*, *HhaI* and *MspI* restriction enzymes for the *BDNF* Val66Met, the *LEP* G2548A, and the *LEPR* Gln223Arg polymorphisms, respectively. Alleles were visualized as fragments by electrophoresis through an ethidium bromide-stained 2.5% agarose gel. Distribution patterns of the *BDNF* Val66Met, the *LEP* G2548A, and the *LEPR* Gln223Arg polymorphisms were shown in Figures 1, 2, 3.

### Statistical analysis

Statistical analysis was performed using SPSS for Windows version 11.5 (SPSS, Chicago, IL). Median, range and 95% confidence intervals (CI) were calculated. The difference between the two groups was compared by Mann-Whitney U/Wilcoxon Rank Sum W test. The differences in genotypic and allelic frequencies of the two groups were assessed by Chi-square test. The Minitab statistical computer program was used to calculate the odds ratio (OR). Spearman rank was used to calculate correlations among the variables. To assess links between MS as a dependent variable and other potential factors, logistic regression was applied. A p-value < 0.05 was considered statistically significant. Goodness-of-fit of the logistic regression models was established by Hosmer-Lemeshow test.

## Results

Anthropometric and biochemical data of the MS and control groups are shown in Table 1. Serum leptin levels were significantly higher in the MS group than the control group (p < 0.01), while lower HDL-C levels were observed in MS group as compared to the control group (p < 0.01). Meanwhile, there were significant differences between the MS and control groups in glucose, insulin, TG, systolic BP, diastolic BP, BMI, waist/hip ratio, WC, AC and TSF (p < 0.01). The BDNF difference between the MS and control groups was not significant. Spearman’s Rank correlation test results are shown in Table 2. Leptin level was positively correlated with age, insulin, TG, systolic BP, diastolic BP, BMI, waist/hip ratio, WC, AC and TSF, but negatively correlated with HDL-C level (p < 0.05). Moreover, BDNF level correlated significantly with age, AC and TSF, as shown in Table 2.

**Table 1:** Comparison of anthropometric, biochemical variables and age between individuals with and without MS

**Variables**	**Control (n = 162)**	**MS (n = 160)**	**p-value**
	**Median (range)**	**Median (range)**	
Age (years)	46.0 (24.0-64.0)	48.0 (28.0-63.0)	0.241
Leptin (ng/mL)	6.2 (2.2-32.3)	11.4 (2.9-36.8)	<0.001**
BDNF (ng/mL)	11.7 (2.8-29.2)	11.5 (2.7-27.8)	0.946
Glucose (mg/dL)	84.0 (66.0-113.0)	95.5 (70.0-134.0)	<0.001**
Insulin (μU/mL)	11.7 (4.5-30.7)	15.8 (5.6-37.3)	<0.001**
TG (mg/dL)	84.0 (58.0-270.0)	169.0 (63.0-302.0)	<0.001**
HDL-C (mg/dL)	50.0 (28.0-77.0)	40.0 (27.0-71.0)	<0.001**
Systolic BP (mmHg)	120.0 (95.0-160.0)	130.0 (100.0-170.0)	<0.001**
Diastolic BP (mmHg)	74.0 (60.0-95.0)	80.0 (64.0-100.0)	<0.001**
BMI (kg/m^2^)	22.8 (18.9-35.5)	26.5 (19.5-37.9)	<0.001**
Waist/Hip ratio	0.82 (0.69-0.97)	0.88 (0.72-0.99)	<0.001**
WC (cm)	77.0 (61.5-101.3)	91.0 (66.5-106.0)	<0.001**
AC (cm)	27.5 (21.8-34.0)	30.2 (22.9-38.5)	<0.001**
TSF (mm)	17.9 (6.5-37.5)	26.0 (8.5-40.9)	<0.001**

*Abbreviation*: *BDNF* Brain-derived neurotrophic factor, *TG* triglyceride, *HDL-C* high-density lipoprotein cholesterol.

*BMI* Body mass index, *WC* waist circumference, *AC* arm circumference, *TSF* triceps skin-fold thickness.

Significant level;** = p < 0.01 by Mann-Whitney U / Wilcoxon Rank Sum W test (2-tailed).

**Table 2:** Correlation coefficients of leptin as well as BDNF with other parameters, in all subjects

**Variables**	**Leptin**	**BDNF**
Leptin	1	0.021
BDNF	0.021	1
Age	0.233**	-0.280**
Glucose	0.097	-0.059
Insulin	0.152*	-0.127
TG	0.236**	0.127
HDL-C	-0.214**	-0.057
Systolic BP	0.358**	-0.018
Diastolic BP	0.199**	-0.015
BMI	0.383**	0.036
Waist/Hip ratio	0.301**	0.027
WC	0.293**	0.052
AC	0.408**	0.314**
TSF	0.516**	0.224**

*Abbreviation*: *BDNF* Brain-derived neurotrophic factor, *TG* triglyceride,

*HDL-C* high-density lipoprotein cholesterol, *BMI* Body mass index, *WC* waist circumference, *AC* arm circumference, *TSF* triceps skin-fold thickness.

Significant levels;* = p < 0.05 by Spearman’s rank correlation (2-tailed).

**= p < 0.01 by Spearman’s rank correlation (2-tailed).

To evaluate the associations of single-nucleotide polymorphisms (SNPs), *LEPR* Gln223Arg, *LEP* G2548A and *BDNF* Val66Met, with leptin level, BDNF level and metabolic features we performed multivariate analysis. Significant associations of *LEPR* Gln223Arg polymorphism were found with leptin and glucose levels, after adjusting for potential covariates including age, sex, weight and smoking status; the results are shown in Table 3. There were no significant associations of *LEP* G2548A and *BDNF* Val66Met polymorphisms with leptin level, BDNF level and metabolic features (p > 0.05); the results are shown in Table 3. The genotypes of SNPs *LEPR* Gln223Arg, *LEP* G2548A and *BDNF* Val66Met were in Hardy-Weinberg equilibrium (HWE) (p > 0.05) and the values of chi-square and degrees of freedom were shown in Table 4. Moreover, the results in genotypic and allelic frequencies of *LEPR* Gln233Arg, *LEP* G2548A, and *BDNF* Val66Met polymorphisms in MS and control subjects are also shown in Table 4. There were significant differences in genotypic and allelic frequencies of *LEPR* Gln233Arg polymorphism between MS and control groups (p < 0.05), but genotypic and allelic distributions of *LEP* G2548A and *BDNF* Val66Met polymorphisms between these two groups were not significantly different (p > 0.05).

**Table 3:** **Adjusted odds ratio for *LEPR *** **Gln223Arg, *LEP *** **G2548A and *BDNF *** **Val66Met polymorphisms with leptin level, BDNF level and metabolic features**

**Variables**	**SNP** ***LEPR*** **Gln223Arg**	**p-value**	**SNP** ***LEP*** **G2548A**	**p-value**	**SNP** ***BDNF*** **Val66Met**	**p-value**
	**OR (95% CI)**		**OR (95% CI)**		**OR (95% CI)**	
Systolic BP	0.989 (0.952-1.027)	0.568	1.030 (0.982-1.090)	0.318	1.022 (0.978-1.068)	0.331
Diastolic BP	1.019 (0.968-1.072)	0.474	1.000 (0.932-1.073)	0.999	1.017 (0.972-1.075)	0.447
WC	0.991 (0.972-1.046)	0.437	1.044 (0.966-1.129)	0.270	0.968 (0.899-1.043)	0.410
TG	0.999 (0.993-1.004)	0.635	1.002 (0.994-1.009)	0.636	0.998 (0.991-1.007)	0.378
HDL-C	1.000 (0.974-1.038)	0.994	0.992 (0.945-1.041)	0.745	1.002 (0.965-1.029)	0.722
Glucose	1.047 (1.004-1.092)	0.033*	1.021 (0.989-1.065)	0.340	0.989 (0.965-1.019)	0.354
Insulin	1.016 (0.997-1.056)	0.099	0.985 (0.944-1.027)	0.473	1.009 (0.972-1.050)	0.600
Leptin	1.192 (1.038-1.379)	0.009**	0.924 (0.816-1.039)	0.209	0.963 (0.873-1.050)	0.903
BDNF	0.997 (0.950-1.045)	0.737	0.986 (0.925-1.051)	0.576	0.980 (0.943-1.045)	0.909

*Abbreviation*: *BDNF* Brain-derived neurotrophic factor, *TG* triglyceride, *HDL-C* high-density lipoprotein cholesterol.

*WC* waist circumference, *LEP* leptin gene, *LEPR* leptin receptor gene, *BDNF* Brain-derived neurotrophic factor gene.

Significant levels;* = p < 0.05, **= p < 0.01.

**Table 4:** **Genotypic and allelic distribution of *LEPR *** **Gln233Arg, *LEP *** **G2548A, and *BDNF *** **Val66Met polymorphisms in MS and control subjects**

	**MS** **n (%)**	**HWE of MS p-value**^**a**^	**Control n (%)**	HWE of control p-value*	**Genotypic or allelic p-value**^**b**^
***LEPR*** **Gln233Arg**					
Genotype					
Arg/Arg	20 (12.5)	0.399	17 (10.5)	0.295	0.044*
Arg/Gln	80 (50.0)	(df = 1)	62 (38.3)	(df = 1)	
Gln/Gln	60 (37.5)	(χ^2^ = 0.71)	83 (51.2)	(χ^2^ = 1.09)	
Allele					
Arg	120 (0.375)		96 (0.296)		0.034*
Gln	200 (0.625)		228 (0.704)		
***LEP*** **G2548A**					
Genotype					
A/A	58 (36.2)	0.638	55 (34.0)	0.483	0.439
A/G	79 (49.4)	(df = 1)	75 (46.3)	(df = 1)	
G/G	23 (14.4)	(χ^2^ = 0.22)	32 (19.7)	(χ^2^ = 0.49)	
Allele					
A	195 (0.609)		185 (0.571)		0.321
G	125 (0.391)		139 (0.429)		
***BDNF*** **Val66Met**					
Genetype					
Met/Met	32 (20.0)	0.479	30 (18.5)	0.544	0.327
Met/Val	84 (52.5)	(df = 1)	75 (46.3)	(df = 1)	
Val/Val	44 (27.5)	(χ^2^ = 0.50)	57 (35.2)	(χ^2^ = 0.37)	
Allele					
Met	148 (0.463)		135 (0.417)		0.241
Val	172 (0.537)		189 (0.583)		

*Abbreviation*: *HWE* Hardy-Weinberg equilibrium, *LEP* leptin gene, *LEPR* leptin receptor gene, *BDNF* Brain-derived neurotrophic factor gene.

Significant level;* = p < 0.05.

^a^Based on the results of chi-square test.

^b^Based on the results of chi-square test for the comparison between MS and control groups.

Association between *LEPR* Gln233Arg, *LEP* G2548A and *BDNF* Val66Met polymorphisms and MS among study subjects are shown in Table 5. Polymorphism of *LEPR* Gln223Arg showed a significant link to MS (OR = 1.8, p < 0.05), and Gln/Arg with Arg/Arg genotype frequencies in the MS and control groups were 62.5% and 48.8%, respectively. In contrast, *LEP* G2548A and *BDNF* Val66Met polymorphisms were not related to MS (p > 0.05).

**Table 5:** **Association between *LEPR *** **Gln233Arg, *LEP *** **G2548A and *BDNF *** **Val66Met polymorphisms and MS**

**SNPs**	**Genotype frequencies**		**OR (95% CI)**	**p-value**^**a**^
	**MS n (%)**	**Control n (%)**		
***LEPR*** **Gln223Arg**				
Genotype				
Arg/Arg + Arg/Gln	100 (62.5)	79 (48.8)	1.8 (1.2-2.7)	0.013*
Gln/Gln	60 (37.5)	83 (51.2)		
***LEP*** **G2548A**				
Genotype				
A/A + A/G	137 (85.6)	130 (80.2)	1.5 (0.8-2.6)	0.200
G/G	23 (14.4)	32 (19.8)		
***BDNF*** **Val66Met**				
Genotype				
Met/Met + Met/Val	116 (72.5)	105 (64.8)	1.4 (0.8-2.5)	0.230
Val/Val	44 (27.5)	57 (35.2)		

*Abbreviation*: *LEP* leptin gene, *LEPR* leptin receptor gene, *BDNF* Brain-derived neurotrophic factor gene.

Significant level;* = p < 0.05.

^a^Based on the results of chi-square test.

Logistic regression results for possible associations between MS and age, sex, TSF, waist/hip ratio, insulin, leptin, *LEP* G2548A, *LEPR* Gln223Arg and *BDNF* Val66Met, are shown in Table 6. Of these variables, leptin, *LEPR* Gln223Arg, TSF and waist/hip ratio were significantly related to MS (p < 0.05). The Hosmer-Lemeshow Goodness-of-Fit test (χ^2^ = 0.896, p > 0.05) was not statistically significant; the fit between the predictive model and the data was acceptable.

**Table 6:** **Logistic regression analysis when MS was used as a dependent variable, and age, sex, TSF, waist/hip ratio, insulin, leptin, *LEPR *** **Gln223Arg, *LEP *** **G2548A and *BDNF *** **Val66Met, were taken as independent variables**

**Variables**	**β**	**p-value**	**OR**	**(95% CI)**
Leptin	0.46	0.007*	1.91	1.25-3.03
*LEPR* Gln223Arg	2.37	0.033*	9.07	1.22-37.14
Waist/hip ratio	5.58	<0.001*	22.04	2.42-91.36
TSF	0.17	0.029*	1.31	1.03-1.95
Sex	-2.34	0.335	0.20	0.02-6.26
Age	0.03	0.625	0.97	0.89-1.14
Insulin	0.07	0.101	1.10	0.98-1.21
*BDNF* Val66Met	0.88	0.179	2.39	0.74-8.54
*LEP* G2548A	1.84	0.124	6.03	0.80-26.11

*Abbreviation*: *LEP* leptin gene, *LEPR* leptin receptor gene, *BDNF* Brain-derived neurotrophic factor gene, *TSF* triceps skin-fold thickness.

Significant level; *= p < 0.05.

## Discussion

To the best of our knowledge, this is the first study to examine the links of leptin level, BDNF level, and polymorphisms of *LEPR* Gln223Arg, *LEP* G2548A and *BDNF* Val66Met with MS among Thais. The key observations of this study were that leptin levels were significantly higher in the MS group than in the control group, and that the *LEPR* Gln223Arg polymorphism showed a significant link to leptin concentrations and MS among Thais. In contrast, *LEP* G2548A and *BDNF* Val66Met polymorphisms were not related to MS.

MS is a cluster of risk factors predictive of future cardiovascular diseases and type 2 diabetes mellitus [1]. Factors which appear to promote the development of MS include genetics, obesity, physical inactivity and an unhealthy diet. Mechanisms causing the onset of this syndrome, however, are still not clearly understood. Leptin is an adipocyte-derived signaling factor that has an important role in metabolic control, such as in stimulating glucose uptake, fatty acid oxidation, and reducing food intake [2,22]. Moreover, circulating leptin levels are closely related to stored adipose tissues [3] and metabolic function [22]. However, most previous studies have focused on the relationship between leptin and obesity, and each component of MS, instead of a cluster of MS components. Therefore, the present study investigated links between leptin and MS among Thais, especially. Our results were in accordance with previous studies in Korean populations [23] and Lebanese volunteers [24], in that serum leptin was associated with MS. Bremer *et al*. also showed increased amounts of subcutaneous adipose tissue secretion and circulating leptin concentrations in subjects with nascent MS [25]. Increased leptin concentration in MS may represent a form of leptin resistance, as in obese subjects [26]; obesity is one of the risk factors for MS. Therefore, alternation of leptin levels may be a part of the etiology of MS.

Leptin exerts a number of significant influences, by binding and activating specific leptin receptors in the hypothalamus and other organs including pancreatic β cells, adipose tissue, and muscle. Previous studies reported *LEPR* polymorphisms were associated with glucose metabolism [27] and insulin resistance [5], possibly leading to a predisposition towards greater MS risk. *LEPR* Gln223Arg polymorphism has been associated with impaired signaling capacity of the leptin receptor [8]. *LEPR* Gln223Arg polymorphism has also been linked with impaired glucose tolerance and conversion to type 2 diabetes [27]. Our study was therefore interested in this position of the *LEPR* gene polymorphism. Present data points to a relationship between *LEPR* Gln223Arg polymorphism and increased leptin and glucose levels in Thai subjects, similar to results in Dutch adults [28] and Romanian subjects [29]. In contrast to our results, however, *LEPR* Gln223Arg polymorphism demonstrated no relationship with leptin levels in Turkish children [11]. Moreover, few studies have determined a correlation between *LEPR* gene polymorphism and MS. Our findings among Thais were in accordance with results from a sample of elderly Brazilian subjects [9], which showed links between *LEPR* Gln223Arg polymorphism and MS. On the other hand, the study in Turkish children did not find any relation between *LEPR* Gln223Arg polymorphism and MS [11]. Therefore, our study demonstrated that *LEPR* Gln223Arg polymorphism in Thai subjects was associated with increased MS risk, which may be explained by increasing glucose and leptin levels. Although the pathogenesis of MS and each of its components is complex and not well understood, our investigation proposes that *LEPR* Gln223Arg polymorphism associated with increased leptin levels may represent a modified functional leptin receptor and may take part in the development of MS. The previous studies also suggested that *LEPR* Gln223Arg polymorphism has been associated with insulin resistance capacity and an altered leptin-binding activity [30] and the leptin can modulates insulin secretion and action via leptin receptors in pancreatic β cells [31].

Although many researches indicate a role for genetic susceptibility to the MS and our results supporting the relationship between the *LEPR* Gln223Arg polymorphism and increased MS risk among Thais, lifestyle factors have also found to be affecting factors in the pathogenesis and progression of MS. Thailand has importantly experienced economic transition and its structure has gradually changed from the traditional agricultural surroundings to an industrialized structure. The dietary intake pattern in Thais has changed from traditional high-carbohydrate diets, which rely heavily on rice and vegetables, to diets high in fat and sugar; physical activity has progressively declined [32]. These transitions have consequently resulted in increasing the prevalence of MS in Thailand [33].Therefore, lifestyle interventions including eating healthy diet and improving physical activity should be regarded as important management for reducing the development of MS among Thais, especially in SNP *LEPR* Gln223Arg subjects. With regard to links between *LEP* G2548A gene polymorphism and leptin levels, the previous study reported a common gene polymorphism in the promoter *LEP* G2548A influencing leptin expression and adipose tissue secretion of leptin [7]. However, studies yielded inconsistent results; data in Egyptian subjects [34] and Romanian subjects [29] points to a relationship between *LEP* 2548GG variants and serum leptin levels, meanwhile studies in Melanesian and Micronesian Solomon Islanders [35] found no relationship between *LEP* G2548A polymorphism and leptin levels, consistent with our results among Thais. In investigating the links between *LEP* G2548A polymorphism and MS, *LEP* G2548A polymorphism was shown to be significantly associated with MS in Tunisian volunteers [10] and Egyptian subjects [34], while no links were found in our study. Investigations among Romanian subjects also found no association of the *LEP* G2548A polymorphism with common obesity-related variables and metabolic traits [29]. It is possible that inconsistencies among the results of the *LEP* and *LEPR* gene polymorphisms in these studies are the result of the different genetic backgrounds or environmental conditions of the populations studied.

BDNF is involved in regulating energy homoeostasis, blood glucose levels, and eating behaviors, and it appears to function downstream of the leptin-melanocortin signaling pathway [13,36]. BDNF is expressed at high levels in the ventromedial hypothalamus (VMH) where its expression is regulated by nutritional state and by melanocortin-4 receptor (MC4R) signaling [36]. Moreover, Komori *et al.* demonstrated that administration of leptin causes an increase in expression of BDNF mRNA and protein in the VMH [37]. The precise mechanism that mediates BDNF expression by leptin remains to be elucidated and there are two evidences that have been proposed. The first possibility is that the LEP- LEPR interaction directly triggers a signal transduction cascade that induces BDNF in the VMH. The second option involves the leptin-mediated production of the melanocortin precursor α-melanocyte stimulating hormone (α-MSH) in the arcuate nucleus, which activates BDNF in the VMH via MC4R [13]. However, the mechanistic implication of the association between leptin, BDNF and the *LEPR* gene in MS is unclear. Our results may imply that Thai MS subjects have related to *LEPR* Gln223Arg polymorphism and increased leptin levels representing a form of leptin resistant. These defects may be involved in the LEP-LEPR interaction in mechanisms induced BDNF expression by leptin signaling. Although previous studies have investigated a correlation between BDNF levels and MS, these investigations have shown inconsistent results of BDNF levels in subjects with MS [16,17,38,39]. In examining links between BDNF concentrations and *BDNF* Val66Met gene polymorphisms with MS among Thais, the present study found neither BDNF concentrations nor *BDNF* gene polymorphism relates to MS. Our results were in line with studies in Spanish and Taiwanese subjects, in that, BDNF concentrations did not correlate with MS and any metabolic syndrome component [16,38]. In contrast, other reports have found BDNF levels to be linked with MS, and with several features of MS [17,39]. However, data on *BDNF* Val66Met gene polymorphism with MS is scarcely available. Indeed, ours is the first publication to determine these associations among Thais. Previous studies found that *BDNF* Val66Met gene polymorphism influenced BDNF expression and/or activity [15], and was related to a higher risk of becoming obese [40]. One study among Caucasian subjects found no significant association between Val66Met gene polymorphism and MS [41]. Similarly, our results confirm this polymorphism not to be linked with MS or with features of MS. Therefore, our findings suggest that BDNF concentrations and *BDNF* Val66Met polymorphism are not involved in the pathophysiology of MS among Thais. However, the relationship between BDNF levels and gene polymorphisms with MS is still not clearly understood, and further studies should be undertaken investigating different ethnicities, as well as other points of the *BDNF* gene.

There are a few limitations of our study. We did not analyze the whole gene of *LEP*, *LEPR* and *BDNF* with MS and further studies should be replicated our findings in larger sample numbers.

In conclusion, the present study found leptin concentrations of MS subjects to be higher than the controls. Although no correlations between MS with BDNF level or *BDNF* Val66Met were observed, there was an association between *LEPR* Gln223Arg polymorphism and leptin level and MS. Therefore, genetic polymorphism in the *LEPR* Gln223Arg appeared to affect the leptin concentration and the susceptibility to MS and these findings might play a significant function in the etiology of MS among the Thai population. Moreover, we believe further studies on MS genetics will help to develop preventive strategies and the role of other genetic and environmental factors should be studied in the other populations.

## Competing interests

The authors have no conflicts of interest to declare.

## Authors’ contributions

KS contributed to the conception and design of the study, collected specimens, carried out laboratory analysis and prepared the manuscript. KT carried out laboratory analysis and helped to draft the manuscript. RT collected specimens and interpretation of the data. All authors read and approved the final manuscript.

## References

[B1] Di Chiara T Argano C Corrao S Scaglione R Licata G Hypoadiponectinemia: a link between visceral obesity and metabolic syndrome J Nutr Metab 2012 2012 175245 22013516 10.1155/2012/175245 PMC3195429

[B2] Poeggeler B Schulz C Pappolla MA Bodó E Tiede S Lehnert H Paus R Leptin and the skin: a new frontier Exp Dermatol 2010 19 12 18 10.1111/j.1600-0625.2009.00930.x 19601981

[B3] Martins M do C Lima Faleiro L Fonseca A Relationship between leptin and body mass and metabolic syndrome in an adult population Rev Port Cardiol 2012 31 711 719 23040870 10.1016/j.repc.2012.08.002

[B4] Duarte SFP Francischetti EA Genelhu-Abreu V Barroso SG Braga JU Cabello PH Pimentel MM p.Q223R leptin receptor polymorphism associated with obesity in Brazilian multiethnic subjects Am J Hum Biol 2006 18 448 453 10.1002/ajhb.20519 16788891

[B5] Chiu KC Chu A Chuang L-M Saad MF Association of leptin receptor polymorphism with insulin resistance Eur J Endocrinol 2004 150 725 729 10.1530/eje.0.1500725 15132731

[B6] Han HR Ryu HJ Cha HS Go MJ Ahn Y Koo BK Cho YM Lee HK Cho NH Shin C Shin HD Kimm K Kim HL Oh B Park KS Genetic variations in the leptin and leptin receptor genes are associated with type 2 diabetes mellitus and metabolic traits in the Korean female population Clin Genet 2008 74 105 115 10.1111/j.1399-0004.2008.01033.x 18564365

[B7] Hoffstedt J Eriksson P Mottagui-Tabar S Arner P A polymorphism in the leptin promoter region (-2548 G/A) influences gene expression and adipose tissue secretion of leptin Horm Metab Res 2002 34 355 359 10.1055/s-2002-33466 12189581

[B8] Yiannakouris N Yannakoulia M Melistas L Chan JL Klimis-Zacas D Mantzoros CS The Q223R polymorphism of the leptin receptor gene is significantly associated with obesity and predicts a small percentage of body weight and body composition variability J Clin Endocrinol Metab 2001 86 4434 4439 11549688 10.1210/jcem.86.9.7842

[B9] Gottlieb MG Bodanese LC Leite LE Schwanke CH Piccoli Jda C da Rocha MI da Cruz IB Association between the Gln223Arg polymorphism of the leptin receptor and metabolic syndrome in free-living community elderly Metab Syndr Relat Disord 2009 7 341 348 10.1089/met.2008.0029 19344216

[B10] Boumaiza I Omezzine A Rejeb J Rebhi L Ouedrani A Ben Rejeb N Nabli N Ben Abdelaziz A Bouslama A Relationship between leptin G2548A and leptin receptor Q223R gene polymorphisms and obesity and metabolic syndrome risk in Tunisian volunteers Genet Test Mol Biomarkers 2012 16 726 733 10.1089/gtmb.2011.0324 22734460 PMC3396002

[B11] Komşu-Ornek Z Demirel F Dursun A Ermiş B Pişkin E Bideci A Leptin receptor gene Gln223Arg polymorphism is not associated with obesity and metabolic syndrome in Turkish children Turk J Pediatr 2012 54 20 24 22397037

[B12] Tapia-Arancibia L Rage F Givalois L Arancibia S Physiology of BDNF: focus on hypothalamic function Front Neuroendocrinol 2004 25 77 107 10.1016/j.yfrne.2004.04.001 15571756

[B13] Rosas-Vargas H Martínez-Ezquerro JD Bienvenu T Brain-derived neurotrophic factor, food intake regulation, and obesity Arch Med Res 2011 42 482 494 10.1016/j.arcmed.2011.09.005 21945389

[B14] Krabbe KS Nielsen AR Krogh-Madsen R Plomgaard P Rasmussen P Erikstrup C Fischer CP Lindegaard B Petersen AM Taudorf S Secher NH Pilegaard H Bruunsgaard H Pedersen BK Brain-derived neurotrophic factor (BDNF) and type 2 diabetes Diabetologia 2007 50 431 438 10.1007/s00125-006-0537-4 17151862

[B15] Chen Z-Y Patel PD Sant G Meng C-X Teng KK Hempstead BL Lee FS Variant brain-derived neurotrophic factor (BDNF) (Met66) alters the intracellular trafficking and activity-dependent secretion of wild-type BDNF in neurosecretory cells and cortical neurons J Neurosci 2004 24 4401 4411 10.1523/JNEUROSCI.0348-04.2004 15128854 PMC6729450

[B16] Lee IT Lee WJ Tsai IC Liang KW Lin SY Wan CJ Fu CP Sheu WH Brain-derived neurotrophic factor not associated with metabolic syndrome but inversely correlated with vascular cell adhesion molecule-1 in men without diabetes Clin Chim Acta 2012 413 944 948 10.1016/j.cca.2012.02.013 22374129

[B17] Chaldakov GN Fiore M Stankulov IS Hristova M Antonelli A Manni L Ghenev PI Angelucci F Aloe L NGF, BDNF, leptin, and mast cells in human coronary atherosclerosis and metabolic syndrome Arch Physiol Biochem 2001 09 357 360 11935372 10.1076/apab.109.4.357.4249

[B18] Jesmin S Islam MR Islam AM Mia MS Sultana SN Zaedi S Yamaguchi N Iwashima Y Hiroe M Watanabe T Comprehensive assessment of metabolic syndrome among rural Bangladeshi women BMC Public Health 2012 12 49 10.1186/1471-2458-12-49 22257743 PMC3293056

[B19] Seltzer CC Goldman RF Mayer J The triceps skinfold as a predictive measure of body density and body fat in obese adolescent girls Pediatrics 1965 36 212 218 14320030

[B20] Collins S Using middle upper arm circumference to assess severe adult malnutrition during famine JAMA 1996 276 391 395 10.1001/jama.1996.03540050051023 8683818

[B21] Matsushita S Kimura M Miyakawa T Yoshino A Murayama M Masaki T Higuchi S Association study of brain-derived neurotrophic factor gene polymorphism and alcoholism Alcohol Clin Exp Res 2004 28 1609 1612 10.1097/01.ALC.0000145697.81741.D2 15547445

[B22] Wauters M Considine RV Van Gaal LF Human leptin: from an adipocyte hormone to an endocrine mediator Eur J Endocrinol 2000 143 293 311 10.1530/eje.0.1430293 11022169

[B23] Yun JE Kimm H Jo J Jee SH Serum leptin is associated with metabolic syndrome in obese and nonobese Korean populations Metabolism 2010 59 424 429 10.1016/j.metabol.2009.08.012 19846168

[B24] Gannagé-Yared M-H Khalife S Semaan M Fares F Jambart S Halaby G Serum adiponectin and leptin levels in relation to the metabolic syndrome, androgenic profile and somatotropic axis in healthy non-diabetic elderly men Eur J Endocrinol 2006 155 167 176 10.1530/eje.1.02175 16793964

[B25] Bremer AA Jialal I Adipose tissue dysfunction in nascent metabolic syndrome J Obes 2013 2013 393192 23653857 10.1155/2013/393192 PMC3638696

[B26] Scarpace PJ Tümer N Peripheral and hypothalamic leptin resistance with age-related obesity Physiol Behav 2001 74 721 727 10.1016/S0031-9384(01)00616-3 11790435

[B27] Salopuro T Pulkkinen L Lindström J Eriksson JG Valle TT Hämäläinen H Ilanne-Parikka P Keinänen-Kiukaanniemi S Tuomilehto J Laakso M Uusitupa M Genetic variation in leptin receptor gene is associated with type 2 diabetes and body weight: the finnish diabetes prevention study Int J Obes 2005 29 1245 1251 10.1038/sj.ijo.0803024 15997246

[B28] Van Rossum CTM Hoebee B van Baak MA Mars M Saris WHM Seidell JC Genetic variation in the leptin receptor gene, leptin, and weight gain in young Dutch adults Obes Res 2003 11 377 386 10.1038/oby.2003.51 12634434

[B29] Constantin A Costache G Sima AV Glavce CS Vladica M Popov DL Leptin G-2548A and leptin receptor Q223R gene polymorphisms are not associated with obesity in Romanian subjects Biochem Biophys Res Commun 2010 391 282 286 10.1016/j.bbrc.2009.11.050 19913498

[B30] Utsunomiya K Shinkai T Sakata S Hwang R Yamada K Chen HI Fukunaka Y Ohmori O Nakamura J Lack of association between the leptin receptor gene (LEPR) Gln223Arg polymorphism and late-onset Alzheimer disease Alzheimer Dis Assoc Disor 2010 24 101 103 10.1097/WAD.0b013e3181b982dc 20220325

[B31] Seufert J Kieffer TJ Leech CA Holz GG Moritz W Ricordi C Habener JF Leptin suppression of insulin secretion and gene expression in human pancreatic islets: implications for the development of adipogenic diabetes mellitus J Clin Endocrinol Metab 1999 84 670 676 10022436 10.1210/jcem.84.2.5460 PMC2927866

[B32] Popkin BM Urbanization, lifestyle changes and the nutrition transition World Dev 1999 27 1905 1916 10.1016/S0305-750X(99)00094-7

[B33] Santibhavank P Prevalence of metabolic syndrome in Nakhon Sawan population J Med Assoc Thai 2007 90 1109 1115 17624204

[B34] Abdel Hay RM Rashed LA Association between the leptin gene 2548G/A polymorphism, the plasma leptin and the metabolic syndrome with psoriasis Exp Dermatol 2011 20 715 719 10.1111/j.1600-0625.2011.01299.x 21569107

[B35] Furusawa T Naka I Yamauchi T Natsuhara K Kimura R Nakazawa M Ishida T Nishida N Eddie R Ohtsuka R Ohashi J The serum leptin level and body mass index in Melanesian and Micronesian Solomon Islanders: focus on genetic factors and urbanization Am J Hum Biol 2011 23 435 444 10.1002/ajhb.21124 21648011

[B36] Xu B Goulding EH Zang K Cepoi D Cone RD Jones KR Tecott LH Reichardt LF Brain-derived neurotrophic factor regulates energy balance downstream of melanocortin-4 receptor Nat Neurosci 2003 6 736 742 10.1038/nn1073 12796784 PMC2710100

[B37] Komori T Morikawa Y Nanjo K Senba E Induction of brain-derived neurotrophic factor by leptin in the ventromedial hypothalamus Neuroscience 2006 139 1107 1115 10.1016/j.neuroscience.2005.12.066 16564638

[B38] Corripio R Gónzalez-Clemente JM Jacobo PS Silvia N Lluis G Joan V Assumpta C Plasma brain-derived neurotrophic factor in prepubertal obese children: results from a 2-year lifestyle intervention programme Clin Endocrinol (Oxf) 2012 77 715 720 10.1111/j.1365-2265.2012.04431.x 22563866

[B39] Golden E Emiliano A Maudsley S Windham BG Carlson OD Egan JM Driscoll I Ferrucci L Martin B Mattson MP Plasma brain-derived neurotrophic factor in prepubertal obese children: results from a 2-year lifestyle intervention programme PLoS One 2010 5 e10099 10.1371/journal.pone.0010099 20404913 PMC2852401

[B40] Beckers S Peeters A Zegers D Mertens I Van Gaal L Van Hul W Association of the BDNF Val66Met variation with obesity in women Mol Genet Metab 2008 95 110 112 10.1016/j.ymgme.2008.06.008 18667348

[B41] Zeman M Jáchymová M Jirák R Vecka M Tvrzická E Stanková B Zák A Polymorphisms of genes for brain-derived neurotrophic factor, methylenetetrahydrofolate reductase, tyrosine hydroxylase, and endothelial nitric oxide synthase in depression and metabolic syndrome Folia Biol (Praha) 2010 56 19 26 20163778 10.14712/fb2010056010019

